# Structural and Serological Characterization of Yet Another New O Antigen, O86, in *Proteus mirabilis* Clinical Strains

**DOI:** 10.3390/ijms252413642

**Published:** 2024-12-20

**Authors:** Dominika Drzewiecka, Evgeniya A. Levina, Alexander S. Shashkov, Nadezhda A. Kalinchuk, Yuriy A. Knirel

**Affiliations:** 1 Department of Biology of Bacteria, Faculty of Biology and Environmental Protection, University of Lodz, 90-237 Lodz, Poland; 2 N. D. Zelinsky Institute of Organic Chemistry, Russian Academy of Sciences, 119991 Moscow, Russia; evgeniya.levina@gmail.com (E.A.L.); alexander.shashkov@mail.ru (A.S.S.); nady_kalinch@mail.ru (N.A.K.); yknirel@gmail.com (Y.A.K.); 3 Higher Chemical College of the Russian Academy of Sciences, D. I. Mendeleev University of Chemical Technology of Russia, 125047 Moscow, Russia

**Keywords:** O-specific polysaccharide, lipopolysaccharide, serotyping, epitope, (*R*)-3-hydroxybutanoic acid, chemical structure

## Abstract

Bacteria from the genus *Proteus* are facultative human pathogens, primarily attacking the urinary tract and wounds. A total of 85 O serogroups have been identified so far among these bacilli. *P. mirabilis* Bprz 86 was isolated from the fistula of a patient in Łódź, Poland. Enzyme-Linked Immunosorbent Assay (ELISA) and Western blotting studies involving the *P. mirabilis* Bprz 86 lipopolysaccharide (LPS) and the strain-specific rabbit antiserum indicated that the strain, which does not belong to any of the O1–O85 serogroups, shares a common epitope with *Proteus* O17 antigens and is identical to another clinical *P. mirabilis* strain, Sm 120, isolated from the urine of a patient in the area. The O-specific polysaccharide (O antigen) was obtained from *P. mirabilis* Bprz 86 LPS through mild acid degradation, and the six-constituent structure of its repeating unit was determined using chemical analyses and 1D and 2D ^1^H and ^13^C Nuclear Magnetic Resonance (NMR) spectroscopy. It includes (*R*)-3-hydroxybutanoyl, which, along with fucosamine and glucose residues, forms a fragment also present in the O17 antigens. Based on the obtained serological and chemical data, the two studied *P. mirabilis* isolates were proposed as candidates for a new successive O serogroup in the genus *Proteus*, O86.

## 1. Introduction

Gram-negative bacilli from the genus *Proteus* belong to the family *Morganellaceae*, which has recently been excluded from the family *Enterobacteriaceae* within the order *Enterobacterales* on the grounds of molecular studies [1]. The bacteria have always been considered potentially dangerous to human and animal health, especially the most pathogenic *Proteus mirabilis* species, which pose a risk of infection mainly to elderly or hospitalized people with compromised immune systems and to children [2]. These opportunistic pathogens are widespread among patients suffering mostly from wound, urinary tract, or gastrointestinal infections [3,4,5]. They are mentioned among the four most common urinary tract pathogens, together with *Escherichia coli*, *Klebsiella pneumoniae*, and *Enterococcus faecalis* [6]. Especially in long-term catheterized patients, they are responsible for 4–5% of catheter-associated urinary tract infections (CAUTIs), which are often recurrent and complicated by biofilm and urinary stone formation [7]. The growing antibiotic resistance observed in many bacteria raises the threat of unsuccessful infection treatment. This is also the case for clinical strains from the genus *Proteus*, a growing number of which are resistant to both β-lactams (including cephalosporines and carbapenems) and non-β-lactam antibiotics (including fluoroquinolones and nitrofurans) [8,9]. What is more, there are no fully effective ways to prevent *Proteus* spp. infections. So far, vaccines composed of heat-killed cells from different uropathogens, including *P. mirabilis* and *P. vulgaris*, are available [6,10]. Moreover, some research has focused on external cell structures that could be potential targets for vaccine development. These are mainly fimbrial and other proteins that play a role in the pathogenesis of *Proteus*, either alone or in mixed infections [2,10]. The bacteria produce many virulence factors, including urease, fimbriae, flagella, toxins, and lipopolysaccharide (LPS) [2]. The polysaccharide part of LPS is the most external part of the cell wall’s outer membrane, contacting the surrounding environment. It is called the O antigen, which is responsible for the serological specificity of *Proteus* spp. strains. O antigens induce the production of specific antibodies in the sera of infected patients or vaccinated animals [11,12]. *Proteus* spp. bacilli are strongly serologically differentiated, with over 80 O serotypes recognized so far, forming 85 O serogroups in the genus [13]. In O antigens from *Proteus* spp. strains, O-repeating units are branched or linear and are composed of three to six monosaccharides. Many of them also contain non-sugar constituents, including amino acids, fatty acids, ethanolamine phosphate, and others. In some O-specific polysaccharides (O-PS), unusual and rare constituents have been detected, such as choline phosphate, malonic acid, and succinic acid [12]. No O serotype has been specifically linked to virulence, although some of them (e.g., O3, O6, O10, O11, O16, O20, O27, O30, and O78) seem to be more frequent in patients [5]. Clinical *P. mirabilis* strains represent at least 50 O serotypes, and *P. mirabilis* strains belonging to new undescribed O serogroups are still being recognized in clinical material. However, to the best of our knowledge, there have been no attempts to use O-antigen epitopes in the development of vaccines preventing *Proteus* spp. infections.

In this work, we serologically and chemically studied the O antigen of the *P. mirabilis* Bprz 86 strain, isolated from a wound. Moreover, we found that another clinical isolate from urine, *P. mirabilis* Sm 120, is serologically identical. These two *P. mirabilis* strains from patients hospitalized in Lodz, Poland, are representatives of a new serogroup, O86, proposed to be created within the genus *Proteus*, sharing a common epitope with the *Proteus* O17 serotype.

## 2. Results and Discussion

### 2.1. Characterization of the Strains

**Figure 1:**
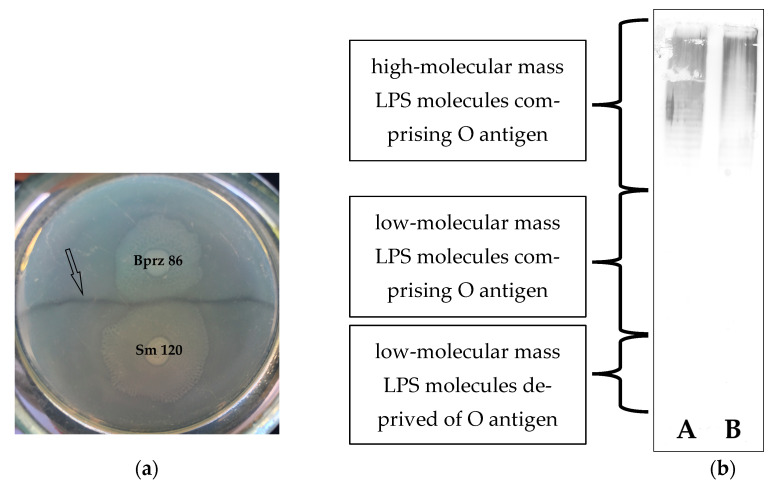
(**a**) Dienes test for the studied *P. mirabilis* Bprz 86 and Sm 120 isolates on nutrient agar plates. The border line (black arrow) is clearly visible between the swarms of the strains; (**b**) Western blot of *P. mirabilis* Bprz 86 lipopolysaccharide (LPS) (A) and *P. mirabilis* Sm 120 biomass (B) with *P. mirabilis* Bprz 86-specific serum.

Clinical isolates Bprz 86 (from wounds) and Sm 120 (from urine) were received in 2009 from two microbiological laboratories in Lodz, Poland, as *P. mirabilis* strains. Their taxonomic position was confirmed by their ability to degrade phenylalanine, urea, and ornithine, whereas mannose and tryptophane were not utilized, using the tests proposed in [14]. We applied the Dienes test to assess the clonal relatedness of the isolates. The Dienes phenomenon is believed to enable reliable differentiation between non-kin *P. mirabilis* strains, which are capable of migrating over solid surfaces (swarming) [15]. The visible border line that forms between the swarms of the isolates indicates that they are not the same clone. On the other hand, the test may also confirm the homology of *P. mirabilis* strains [16]. As a result of the experiment, the visible line of demarcation (the Dienes line) formed between the mutually inhibited swarming growth of the two studied strains, and their swarms did not merge on the nutrient broth-agar plate (Figure 1a). The results clearly suggest that the isolates are not kin related.

### 2.2. Serological Studies

Good swarming abilities confirmed that both studied *P. mirabilis* strains produce full-length LPS comprising the O polysaccharide (OPS) (the O antigen) and thus enabling a good embedding of the flagella in the outer membrane. To determine the O serotype (the O-antigen type) of the studied isolates, in the first step, the biomasses of the *P. mirabilis* Bprz 86 and *P. mirabilis* Sm 120 strains were examined with a set of rabbit antisera specific to the recognized O serotypes in the genus *Proteus*, using the Enzyme-Linked Immunsorbent Assay (ELISA). However, the O antigens present on the cells of both strains were not recognized by any of the antisera on a level comparable to that in the reactions in homologous systems (titers 1:320,000–1:1,024,000). Therefore, in the next step, the LPS was extracted from *P. mirabilis* Bprz 86 dry biomass (with the yield of 6.15%) and a rabbit O-specific serum was obtained for this strain. Again, the LPS and the O-antiserum did not react in ELISA with O1–O85 antisera/LPSs, respectively, or the reactions were not strong enough to give a basis for classifying of the Bprz 86 strain to one of the O serogroups in the genus *Proteus*.

The only strong reaction of the obtained *P. mirabilis* Bprz 86 antiserum was observed with the biomass of the second studied clinical strain, *P. mirabilis* Sm 120, which was also recognized as not belonging to any of the described O1–O85 serogroups in the genus *Proteus*. The observed cross reaction of the *P. mirabilis* Sm120 cells was as strong as the reaction of the *P. mirabilis* Bprz 86-specific serum in the homologous system (Table 1).

Thus, the *P. mirabilis* Sm 120 isolate was acknowledged to be serologically unique but, at the same time, related to the *P. mirabilis* Bprz 86 strain. In order to assess the degree of the strains’ similarity, we used the adsorption technique. When the *P. mirabilis* Bprz 86 antiserum was adsorbed by the *P. mirabilis* Sm 120 cells, all the antibodies specific to the Bprz 86 strain were removed by the adsorbing strain, as no reaction of the adsorbed Bprz 86 antiserum was detected in the homologous system (with Bprz 86 LPS) (Table 1), which confirmed the serological identity of both isolates. At the same time, the lack of reaction with the *P. mirabilis* Sm 120 biomass proved that the adsorption process was effective. The results of the Western blotting technique revealed that both studied strains produced long-chained LPS and that the Bprz 86-specific serum lacked core-specific antibodies. For both strains, the reactions were visible only with the slowly migrating LPS bands, which comprised the OPS part (the full length LPS molecules) (Figure 1b). The serological identity of the studied *P. mirabilis* strains Bprz 86 and Sm 120 might indicate that they spread between patients in the area. However, the studied strains were assumed to be various clones coming from different patients but, at the same time, possessing identical O antigens.

Although the serologically unique *P. mirabilis* strains Bprz 86 and Sm 120 proved to be unclassifiable according to the current serological classification scheme in the genus *Proteus*, some weak cross reactions of the *P. mirabilis* Bprz 86-specific serum were observed in ELISA. However, the antiserum recognized only the LPSs of the strains representing the O17 serogroup, e.g., *P. mirabilis* PrK 32/57 and *P. vulgaris* 33/57 [17] as well as *P. penneri* 20 [18] (Table 1). The reactions in the opposite system (*P. mirabilis* Bprz 86 LPS with the O17-specific sera) were a little stronger (Table 2), indicating some serological similarities between the OPSproduced by the studied *P. mirabilis* Bprz 86 isolate and the O17 antigens.

To confirm this observation, the O17 antisera were adsorbed using the cells of the studied cross-reacting *P. mirabilis* Bprz 86 strain. As a result, some fractions of antibodies were removed from the antisera, and the ELISA reaction titers of the O17 sera with the homologous LPSs were decreased (Table 2). During the adsorption process, the *P. mirabilis* Bprz 86 biomass bound all the antibodies present in the O17 antisera which were recognizing the *P. mirabilis* Bprz 86 LPS (titers < 1000). It confirmed that the adsorption process had been carried out effectively and properly. These results clearly indicate that the O17 antigens and the studied *P. mirabilis* Bprz 86 OPS display serological similarities, i.e., that they share common epitope(s), due to the probably identical structural fragment(s) in their O repeating units. Thus, in the respective O-antisera, there are fractions of antibodies showing the same specificity and recognizing the same epitope present both in the O17 and Bprz 86 O antigens. Cross adsorption was found to deprive the antisera of the common antibodies fractions, thus decreasing their reactivities in the homologous systems, which confirmed the mutual similarity but not identity of the *P. mirabilis* Bprz 86 and *Proteus* O17 antigens.

### 2.3. Structural Studies

A long-chain OPS was obtained by mild acid degradation of the LPS of the *P. mirabilis* Bprz 86 strain followed by gel permeation chromatography (GPC) on Sephadex G-50 Superfine. Sugar analysis by gas-liquid chromatography (GLC) and gas–liquid chromatography-mass spectrometry (GLC-MS) of the acetylated alditols derived from the OPS by acid hydrolysis followed by borohydride reduction [19] revealed Glc, GlcNAc, GalNAc, Fuc3NAc, and Fuc3NHb, where Hb indicates 3-hydroxybutanoyl; the ratios of the monosaccharide derivatives were, respectively, 2:0.77:0.72:0.40:0.45 (detector response). Further studies showed that the OPS contained Fuc3NHb only, and the appearance of Fuc3NAc was evidently due to a partial removal of the Hb group in the course of acid hydrolysis followed by N-acetylation of the released Fuc3N. GLC of the acetylated (+)-2-octyl glycosides [20] and trifluoroacetylated (+)-2-octyl esters [21] indicated the d configuration of all constituent monosaccharides and the *R* configuration of 3-hydroxybutanoic acid, respectively.

**Figure 2:**
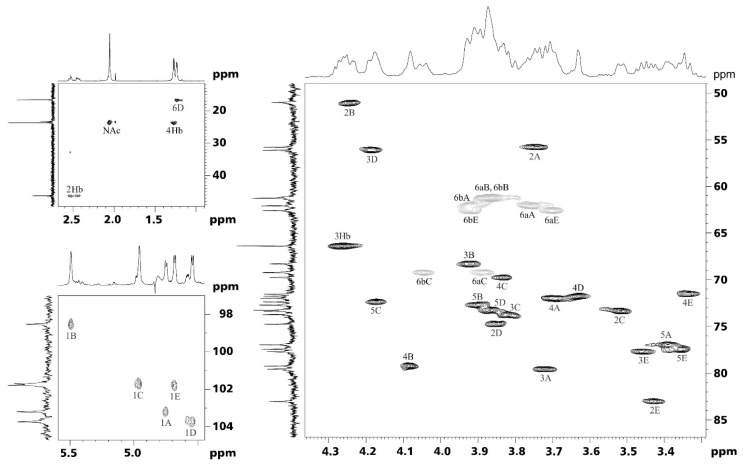
Parts of a ^1^H,^13^C Heteronuclear Single Quantum Coherence (HSQC) spectrum of the O polysaccharide (OPS) of the *P. mirabilis* Bprz 86 strain. The corresponding parts of the ^1^H and ^13^C Nuclear Magnetic Resonance (NMR) spectra are shown along the horizontal and vertical axes, respectively. The numbers refer to H/C pairs in sugar residues denoted by letters as indicated in Table 3 and Figure 3a.

The ^13^C Nuclear Magnetic Resonance (NMR) spectrum of the OPS (Figure 2) showed signals for five anomeric carbons at δ 98.5–103.7, one *C*H_3_-C group (C-6 of Fuc3N) at δ 16.7, four O*C*H_2_-C groups (C-6 of Glc, GlcN, and GalN) at δ 61.2–62.5 and 66.2, three nitrogen-bearing carbons (C-2 of GlcN and GalN, C-3 of Fuc3N) at δ 51.0–56.1, non-anomeric oxygen-linked carbons at δ 68.3–80.3, two *N*-acetyl groups at δ 23.7 (2 CH_3_), one *N*-(3-hydroxybutanoyl) group at δ 23.4 (C-4), 46.2 (C-2) and 66.4 (C-3), and CO groups at δ 174.8–175.8 (Table 3). Accordingly, the ^1^H NMR spectrum of the OPS (Figure 2) contained signals for five anomeric protons at δ 4.54–5.45, one CH_3_-C group (H-6 of Fuc3N) at δ 1.24, other sugar protons at δ 3.34–4.25, two N-acetyl groups at δ 2.06 (2 CH_3_), and one *N*-(3-hydroxybutanoyl) group at δ 1.27 (H-4), 2.44, 2.53 (H-2a,2b), and 4.27 (H-3) (Table 3).

The NMR spectra of the OPS were assigned using ^1^H,^1^H correlation spectroscopy (COSY), ^1^H,^1^H total correlation spectroscopy (TOCSY), and ^1^H,^13^C Heteronuclear Single Quantum Coherence (HSQC) (Figure 2) experiments, and spin systems for α-Glc*p* (unit C), β-Glc*p* (unit E), β-Glc*p*NAc (unit A), α-Gal*p*NAc (unit B), and β-Fuc*p*3Hb (unit D) were identified (Table 3). The 2D ^1^H,^1^H rotating-frame nuclear Overhauser effect spectroscopy (ROESY) spectrum of the OPS showed the following correlations between the anomeric protons and protons at the linkage carbons: A H-1/E H-2, E H-1/D H-2, C H-1/B H-4, and B H-1/A H-4 at δ 4.75/3.42, 4.68/3.85, 4.96/4.08, and 5.45/3.72, respectively. These data defined the linkages and the sequence of the monosaccharides in the repeating unit of the OPS. There was no clear correlation for D H-1 but a low-field position of the signal for C-6 of unit C at δ 66.2 in the ^13^C NMR spectrum of the OPS, as compared with its positions in the spectrum of α-Glc*p* at δ 61.9 [21], indicated substitution of unit D at position 6 and thus defined a β-d-Fuc*p*3N*R*Hb-(1→6)-d-Glc*p* (D→C) linkage. Similarly, the positions of glycosylation of the other constituent monosaccharides were confirmed by low-field positions of the signals for the linkage carbons (Table 3), as compared with their positions in the spectra of the corresponding non-substituted monosaccharides [21].

**Figure 3:**
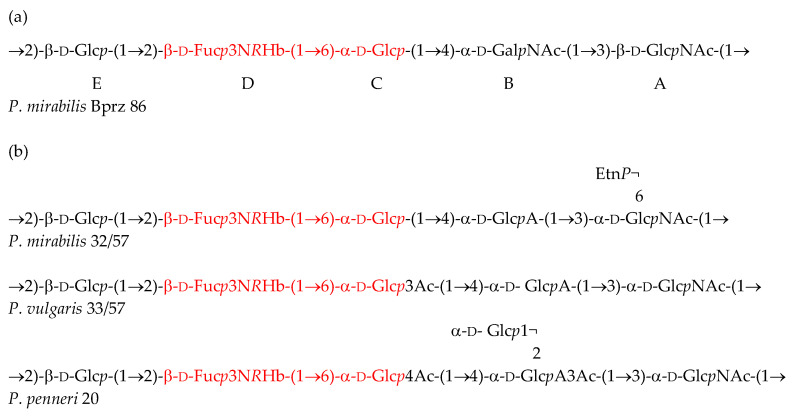
The O-repeating unit chemical structure of (**a**) the *P. mirabilis* Bprz 86 studied strain and (**b**) the representatives of the O17 serogroup [12]. The shared fragment forming the common serological epitope is marked in red.

Therefore, it can be stated that the OPS of the *P. mirabilis* Bprz 86 strain has the following structure (Figure 3a):

A peculiar feature of this polysaccharide is the presence of Fuc3N*R*Hb. Although not common in nature, this monosaccharide has been found in the surface polysaccharides of various bacteria (Bacterial Carbohydrate Structure DataBase at http://csdb.glycoscience.ru/bacterial/ (accessed on 22 October 2024)), including OPSs of several *Proteus* strains classified into serogroup O17 [12].

### 2.4. Classification of the Studied Strains

The studied *P. mirabilis* Bprz 86 and Sm 120 clinical isolates coming from two patients represent different clones in the species *P. mirabilis*. However, they bear the same O antigen, which may be a result of the strains’ adaptation to varying conditions during the passage between human hosts. The strains proved to be serologically identical and, at the same time, unique, as they could not be classified to any of the O1–O85 serogroups known so far in the genus *Proteus*. Moreover, the studied Bprz 86-strain OPShas a structure different from the other studied *Proteus* O antigens [13]. Therefore, we propose that both studied strains be classified to a newly created O86 serogroup in the genus *Proteus*. The genus *Proteus* seems to be one of the most differentiated among the bacilli belonging to the order *Enterobacterales*, covering the previous family *Enterobacteriaceae* [1], as compared to other genera, such as *Salmonella* and *Shigella* (each 46 O serotypes), *Yersinia* (35), *Citrobacter* (43), *Hafnia* (39), or several *Klebsiella*. Among the *Enterobaterales*, only the species *Escherichia coli* includes more serogroups (180) and the species *Vibrio cholerae* (200), which is outside the order [22].

Although there are some similarities in the structures of the new O86 antigen and several other *Proteus* O serotypes, no cross reactivity was noticed except for weak mutual reactions between the studied *P. mirabilis* O86 LPS/antiserum with *Proteus* O17 antisera/LPSs, respectively. The slightly differing O17 polysaccharides possess in their structures the unique monosaccharide Fuc3N*R*Hb linked to the residue of α-d-Glc*p* by a (1→6) linkage (Figure 3b) [12], which is present also in the studied O86 antigen (Figure 3a). The backbone polysaccharide common to all the O17 serotypes has been indicated as a major epitope, playing the main role in the specificity of O17 antigens. However, other epitopes were also suggested to be involved in the serological specificity of different subserotypes, e.g., those associated with ethanolamine phosphate in *P. mirabilis* PrK 32/57, side-branched glucose in *P. penneri* O17 strains or with *O*-acetyl groups [17,18] (which are absent in *P. mirabilis* PrK 32/57 and *P. mirabilis* Bprz 86). In this work, we indicate that the β-d-Fuc*p*3N*R*Hb-(1→6)-α-d-Glc*p* disaccharide including the rare and unique constituent Fuc3N*R*Hb plays the role of a common epitope of minor importance in the O86 antigen and O17 serotypes. It is also important to note that d-Fuc*p*3N or *R*Hb alone did not cause the production of specific antibodies in the studied antiserum, as no cross reactions were noticed between the new O86 serotype and O45 one (comprising d-Fuc*p*3N) or O4 one (comprising *R*Hb) [12].

A serological and structural investigation of the O86 antigen is the next necessary step toward extending the serological classification of the genus *Proteus*. Knowledge of the incidence of particular O serotypes in patients, as well as the similarities between them (common epitopes responsible for cross-reactions), may be helpful in the research on the use of OPSs in the development of vaccines preventing *Proteus* spp. infections. Such studies are being successfully conducted in relation to other members of *Enterobacterales* using the techniques based on bioconjugates of O antigens with protein carriers [23]. Among them, many reports are being published on positive results in clinical trials, mostly with the anti-*Shigella* spp., *-E. coli*, and -*K. pneumoniae* bioconjugative vaccines [24,25,26,27,28,29]. Due to the lack of an effective anti-*Proteus* vaccine, the use of well-defined *Proteus* OPSs may be a promising alternative in the development of preparations protecting against infections caused by these facultative pathogens. It may be also possible to find common epitopes shared by the O antigens in *Proteus* and other uropathogenic genera, e.g., *Klebsiella* [30].

## 3. Materials and Methods

### 3.1. Bacterial Strains, Cultivation of Bacteria, Preparation of Antiserum

*P. mirabilis* strain Bprz 86 was isolated from a fistula of a 54-year-old female patient in November 2009, and *P. mirabilis* strain Sm 120 was isolated from the urine of a 22-year-old female patient in August 2009. Both patients were hospitalized in Lodz, Poland. The isolates were kindly provided by the microbiological laboratory in the Barlicki Hospital in Lodz, Poland and by the Synevo Medical Laboratory in Lodz, Poland, respectively. The strains were stored with the addition of 25% glycerol at −80 °C.

The kinship of the two strains was examined using the Dienes test [15]. The strains were spot inoculated on an agar nutrient broth plate. After 24-h incubation at 37 °C, the presence of a visible border line between the swarming growth of the strains was observed.

The biomass of the strains was obtained by 18-h cultivation in an aerated broth culture (BTL, Lodz, Poland) supplemented with 0.2% of glucose, killed by the addition of 1% (*v*/*v*) phenol, centrifuged, and washed in distilled water.

The rabbit polyclonal antiserum was obtained by a three-time (each five days), 18-day intravenous vaccination with heat-killed Bprz 86 cells from the 1.5 × 10^10^ suspension (the approval of The Ninth Local Ethical Committee on Animal Testing in Lodz, from 17 July 2006, permission number 29/ŁB333/2006).

Adsorptions of 1:100 diluted O17 antisera with the biomass of the cross-reacting *P. mirabilis* Bprz 86 strain (in the *v*/*v* ratio 10:1) were done in three replicates [5].

The rabbit polyclonal O1–O85 antisera and the LPS samples, representing the O serotypes described in the genus *Proteus*, belong to the Department of Biology of Bacteria, University of Lodz, Lodz, Poland.

### 3.2. Isolation of Lipopolysaccharide (LPS) and O-Specific Polysaccharide (OPS) Samples

The LPSs of both studied strains were extracted from the dried (lyophilized) biomass using the phenol-water method [31]. Briefly, 20 g of a bacterial mass was added to 500 mL of 45% phenol aqueous (aq.) solution and heated at 67 °C for 5 min, then cooled. The residual biomass was removed by centrifugation and the extracted LPS solution was purified from phenol by a dialysis against tap and distilled water. The LPS was thickened by water evaporation and purified by precipitation of contaminants by adding aq 50% trichloroacetic acid to the final pH 2.5. The sediment was removed by centrifugation, the acid by dialysis, and the LPS was lyophilized.

An LPS sample (50 mg) was hydrolyzed with aq. 2% HOAc (4 mL) at 100 °C for 2 h, a lipid precipitate was removed by centrifugation (13,000× *g*, 20 min), and an OPS sample was obtained by fractionation of the supernatant by GPC on a column (60 × 2.5 cm) of Sephadex G-50 Superfine (Amersham Biosciences, Uppsala, Sweden) in 0.05 M pyridinium acetate buffer pH 4.5 monitored with a differential refractometer (Knauer, Berlin, Germany).

### 3.3. Serological Techniques

ELISA was performed according to the method described in [5]. The LPS samples or biomasses (antigens) were coated on the ELISA F96 Maxisorp Nunc-Immuno plates (Thermo Fisher Scientific, Waltham, MA, USA) in the amounts of 50 ng or 5 μg per well, respectively. The O-specific antisera were serially diluted (q = 2) starting from 1:1000. The rabbit-IgG specific peroxidase-conjugated goat antibodies (Jackson ImmunoResearch, West Grove, PA, USA) were applied to recognize the serum antibodies bound to the LPS molecules. Additionally, 2,2′-Azino-bis(3-ethylbenzothiazoline-6-sulfonic acid) diammonium salt (Sigma, St. Louis, MI, USA) with 0.1% H_2_O_2_ was used as the peroxidase substrate, and the resulting color reaction (absorbance) was read at 405 nm in a Multiscan Go microplate reader (Thermo Fisher Scientific, Waltham, MA, USA). Absorbance ≥ 0.2 was considered as a positive reaction, while the last antiserum dilution yielding the positive reaction was defined as a titer.

In Western blotting [5], 6 μg of the studied LPSs or proteinase K-treated biomass was added per lane to sodium dodecyl sulphate (SDS)-polyacrylamide gel and electrophoretically fractionated. The separated LPS molecules were transferred to nitrocellulose. Then, the cross-reacting rabbit antiserum was added and the reactions of the serum antibodies with LPS molecules were visualized by the application of goat anti-rabbit-IgG antibodies conjugated with alkaline phosphatase (Jackson ImmunoResearch, West Grove, PA, USA) and a proper AP Conjugate Substrate Kit (Bio-Rad, Hercules, CA, USA).

### 3.4. Composition Analysis

For sugar analysis, an OPS sample (0.5 mg) was subjected to acid hydrolysis (2 M CF_3_CO_2_H, 120 °C, 2 h) followed by a reduction with an excess of NaBH_4_ (20 °C, 2 h), acetylation with a 1:1 (*v*/*v*) Ac_2_O-pyridine mixture (100 °C, 1 h), and analysis by GLC on an Agilent 7820A GC System (Maestro, Moscow, Russia) equipped with an HP-5ms column (Agilent) using a temperature gradient of 7 °C min^−1^ from 160 to 290 °C. The absolute configurations of monosaccharides and 3-hydroxybutanoic acid were determined as described [20,21].

### 3.5. Nuclear Magnetic Resonance (NMR) Spectroscopy

NMR spectra were recorded in 99.95% D_2_O (after deuterium-exchange by freeze-drying from 99.9% D_2_O) at 20 °C using a Bruker Avance II 600 MHz spectrometer and the Bruker TopSpin 2.1 program for acquiring and processing the NMR data. Internal sodium 3-trimethylsilylpropanoate-2,2,3,3-d_4_ (δ_H_ 0, δ_C_ −1.6) was used as reference for calibration. A mixing time of 200 and 150 ms was used in 2D TOCSY and ROESY experiments, respectively.

## Figures and Tables

**Table 1:** Reactivity (reciprocal titers) of the *P. mirabilis* Bprz 86 antiserum (native and adsorbed) with the respective LPSs, in Enzyme-Linked Immunosorbent Assay (ELISA).

Bprz 86 Antiserum	LPS				
	*Pm* Bprz 86	*Pm* Sm 120	*Pm* 32/57	*Pv* 33/57	*Pp* 20
native	*512,000*	512,000	2000	8000	4000
adsorbed with Sm 120	<1000	<1000	ns	ns	ns

ns—not studied; *Pm*—*P. mirabilis*; *Pv*—*P. vulgaris*; *Pp*—*P. penneri*; the reaction in the homologous system is italicized.

**Table 2:** Reactivity (reciprocal titers) of the O17 antisera (native and adsorbed with *P. mirabilis* Bprz 86 cells) with *P. mirabilis* Bprz 86 LPS and O17 LPSs homologous to individual antisera, in ELISA.

LPS	Antiserum					
	*Pm* 32/57		*Pv* 33/57		*Pp* 20	
	Native	Adsorbed	Native	Adsorbed	Native	Adsorbed
Bprz 86	4000	<1000	16,000	<1000	32/64,000	<1000
homologous to antiserum	*512,000*	128,000	*512,000*	64,000	*512,000*	64,000

*Pm*—*P. mirabilis*; *Pv*—*P. vulgaris*; *Pp*—*P. penneri*; the reactions in homologous systems are italicized.

**Table 3:** ^1^H and ^13^C NMR chemical shifts (δ, ppm) of the O-specific polysaccharide of the *P. mirabilis* Bprz 86 strain.

Monosaccharide Residue	C-1 *H-1*	C-2 *H-2*	C-3 *H-3*	C-4 *H-4*	C-5 *H-5*	C-6 *H-6 (6a,6b)*
→3)-β-d-Glc*p*NAc-(1→ A	103.2	55.8	79.6	72.0	77.0	62.2
	*4.75*	*3.74*	*3.72*	*3.70*	*3.39*	*3.76, 3.92*
→4)-α-d-Gal*p*NAc-(1→ B	98.5	51.0	68.3	79.2	72.7	61.2
	*5.45*	*4.25*	*3.92*	*4.08*	*3.90*	*3.85, 3.89*
→6)-α-d-Glc*p*-(1→ C	101.7	73.4	73.8	69.8	72.4	66.2
	*4.96*	*3.52*	*3.83*	*3.83*	*4.17*	*3.83, 4.05*
→2)-β-d-Fuc*p*3N*R*Hb-(1→ D	103.7	74.7	56.1	71.8	73.2	16.7
	*4.54*	*3.85*	*4.19*	*3.63*	*3.87*	*1.24*
→2)-β-d-Glc*p*-(1→ E	101.8	80.3	77.7	71.5	77.4	62.5
	*4.68*	*3.42*	*3.46*	*3.34*	*3.35*	3.70, 3.92

^1^H NMR chemical shifts are italicized. Chemical shifts for the *N*-acetyl groups are δ_H_ 2.06 (2 CH_3_), δ_C_ 23.7 (2 CH_3_), 175.6, and 175.8 (both CO); for the *N*-(3-hydroxybutanoyl) group δ_H_ 1.27 (H-4), 2.44, 2.53 (H-2a,2b), 4.27 (H-3), δ_C_ 174.8, 46.2, 66.4, and 23.4 (C-1–C-4, respectively).

## Data Availability

The data that support the findings of this study are available from the corresponding author on reasonable request.
